# Vector Integration Sites Identification for Gene-Trap Screening in Mammalian Haploid Cells

**DOI:** 10.1038/srep44736

**Published:** 2017-03-17

**Authors:** Jian Yu, Constance Ciaudo

**Affiliations:** 1 Swiss Federal Institute of Technology Zurich, Department of Biology, Institute of Molecular Health Sciences, Chair of RNAi and Genome Integrity, Zurich, Switzerland; 2 Life Science Zurich Graduate School, Molecular and Translational Biomedicine program, University of Zurich, Zurich, Switzerland

## Abstract

Forward genetic screens using retroviral (or transposon) gene-trap vectors in a haploid genome revolutionized the investigation of molecular networks in mammals. However, the sequencing data generated by Phenotypic interrogation followed by Tag sequencing (PhiT-seq) were not well characterized. The analysis of human and mouse haploid screens allowed us to describe PhiT-seq data and to define quality control steps. Moreover, we identified several blind spots in both haploid genomes where gene-trap vectors can hardly integrate. Integration of transcriptomic data improved the performance of candidate gene identification. Furthermore, we experimented with various statistical tests to account for biological replicates in PhiT-seq and investigated the effect of normalization methods and other parameters on the performance. Finally, we developed: VISITs, a dedicated pipeline for analyzing PhiT-seq data (https://sourceforge.net/projects/visits/).

Forward genetic screens using retroviral (or transposon) gene-trap vectors have opened the doors for the investigation of molecular circuitries responsible for various biological processes^1^. Starting with yeast, a significant portion of the knowledge in modern biology is built on hypotheses originated from unbiased screens. In mammalian cells, this approach has been enabled with the establishment of haploid cells from human^2^ and mouse^3^ organisms. After using phenotypic interrogation via tag sequencing (PhiT-seq) in a haploid genome, researchers are now able to produce a reliable genome-wide overview of genes involved in their phenotypes of interest. These studies include gene essentiality^4^,^5^, different biological processes^6^,^7^,^8^, diseases mechanisms^9^,^10^ and stem cells exit from pluripotency^11^.

Nowadays, PhiT-seq data have not been characterized in depth. Moreover, some basic and important questions remain unanswered, including: how to determine the quality of the data; and how many genomic elements PhiT-seq data could cover? Additionally, there is no dedicated bioinformatics pipeline to analyze and visualize these data. Computational frameworks have been developed for the analysis of transposon insertion sequencing (Tn-Seq) for essentiality studies in prokaryotes^12^,^13^,^14^ using sliding-window approaches. These methods cannot be generalized to PhiT-seq due to the tremendous difference in the genome size between prokaryotes and mammals, leading to a lower coverage of sequencing and an insufficient power of the sliding-window approach^15^. The complexity of the architecture of mammalian genomes determines if the insertion site of the vector has to be treated differently. Indeed, an insertion in the antisense orientation of an intronic region will not have the same effect as an insertion in an exonic region. Some computational tools, designed for insertional mutagenesis screens (IMS) of tumorigenesis studies, like TAPDANCE^16^ and PRIM^17^ have also been developed. Nevertheless, PhiT-seq data differ considerably from IMS data in both experiment design and purpose. For example, to account for tumor heterogeneity, the data used in IMS always contain multiple samples, to identify common insertion sites involved in tumor formation^18^, while PhiT-seq aims to identify mutation sites enriched with high-density insertions in selected compared to control samples^10^. Therefore, both algorithms developed for IMS and Tn-seq cannot be directly implemented to analyze PhiT-seq data.

In previous publications where PhiT-seq experiments were conducted in human and mouse haploid cells, in-house methods (proximity index, Fisher’s exact and the binomial tests) were used for the statistical analysis^4^,^9^,^10^,^11^,^19^; however, none of them were packaged into a functional pipeline with other necessary steps, e.g., pre-processing, quality control and visualization. Additionally, these methods were not optimized for mammalian gene structures, leading to a potential loss of information. More importantly, with the introduction of biological replicates and the paired nature of PhiT-seq experiments (control vs. selected), more complex experimental designs have to be supported.

In this study, we first introduced several measurements in order to evaluate the quality of PhiT-seq data and defined blind spots of the screening experiment by using two datasets from human and mouse haploid cells^7^,^10^. To fully exploit the genome structure of mammalian cells, gene models were recompiled by integrating transcriptomic data, increasing the performance for the identification of candidate genes. Several existing frameworks for statistical analysis were evaluated, and their usage was adapted to PhiT-seq experiments. We also investigated the effects of duplicated reads on the results, and compared different normalization methods used in the analysis of different omics data. Subsequently, candidate genes were prioritized using a combined score, which demonstrated increased performance on identifying known genes as well as the capability to reveal novel candidate genes. Finally, we presented VISITs (Vector Integration Sites Identification from PhiT-seq), a dedicated pipeline for the analysis of PhiT-seq data.

## Results and Discussion

### Datasets collection

Two datasets from human and mouse were used in this study^7^,^10^. The first dataset originated from a human PhiT-seq experiment aiming to identify genes involved in Lassa virus entry^10^ using HAP1 cells (referred to as ‘the human dataset’). This dataset was composed of one control library and one selected library (the other two selected libraries were mutants, and were only used in the quality control step). Two single-end RNA-sequencing (RNA-seq) libraries supplemented this dataset^20^. The second dataset was derived from a mouse PhiT-seq experiment aiming to decipher novel genes regulating X-chromosome inactivation (XCI)^7^, using haploid mouse embryonic stem cells (referred to as ‘the mouse dataset’). It included six paired replicates for both control and selected libraries.

### Pre-processing and quality control (QC)

It is widely accepted that pre-processing and QC are crucial steps in Next Generation Sequencing (NGS) data analysis^21^,^22^. However, no analytic tools and software dedicated to PhiT-seq data are publicly available. In this study, we started with pre-processing of aligned PhiT-seq data and proposed several QC measurements for the screening experiment. We assumed that reads trimming, adapter removal and alignment of raw reads had already been properly performed by generic tools, e.g., Trimmomatic^23^ and bowtie^24^. We described pre-processing and QC together, as in practice these two steps are coupled, i.e., QC, pre-processing, and QC again^22^.

In the pre-processing step, reads with multiple-hit positions in the genome were removed and only one insertion site of the duplicated reads (reads with same starting site and orientation) was kept for downstream analysis. All the reads mapping the ChrY were also discarded as most of the current mammalian haploid cells used for screening lack this chromosome, e.g., human HAP1 cells and mouse parthenogenetic haploid embryonic stem cells^3^,^25^. Indeed, we reasoned that any reads mapping to the ChrY were due to sequencing errors or repeated sequences in pseudo-autosomal regions^26^,^27^, and should be removed.

**Figure 1:**
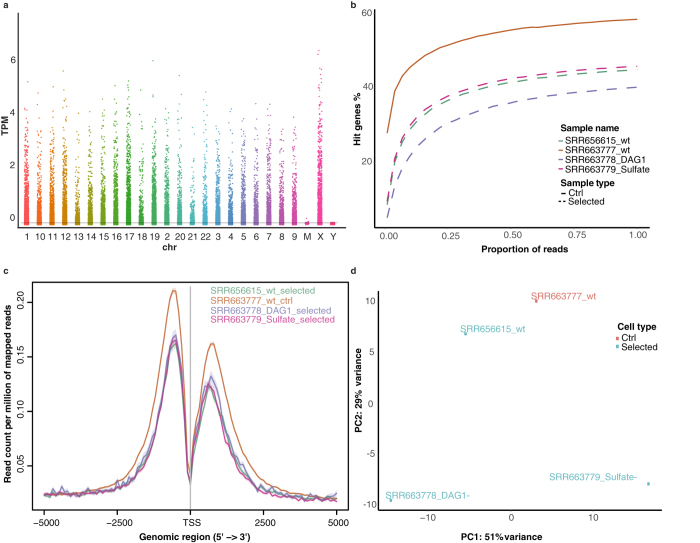
Quality control (QC) of the human dataset in VISITs, including (**a**) Manhattan plot showing vector insertion densities across all chromosomes in the control library. TPM: Transcripts per Million reads. (**b**) Saturation curves for both control and selected libraries. (**c**) Insertion profile near transcription starting sites (TSS) for both control and selected libraries after removing duplicates. (**d**) PCA plot showing sample similarities.

In addition to the classical FastQC^28^ checking for the sequencing quality, we specifically focused on the assessment of the entire screening experiment. The output of its QC was illustrated using the human dataset after pre-processing, including: Manhattan plot, saturation curve, insertion profile and principle component analysis (PCA) plot (Fig. 1). The Manhattan plot aimed to visualize the landscape of vector insertions in the genome of the haploid cells (Fig. 1a). Few insertions should be located on the ChrY before pre-processing and all of them should be mapped on autosomes and X chromosome after pre-processing.

The saturation curve determined if the sequencing depth of each library was sufficient in terms of gene coverage (Fig. 1b). With the increase of reads resampled, the curve should increase at first and reach saturation at the end. Curves without this pattern indicated an unsaturated sequencing, which could lead to a decrease in sensitivity and specificity in the downstream analysis. Saturation curves also informed about the efficiency of the screen. Only the clonal progeny of cells carrying insertions in certain genes should be recovered from a successful screen. Consequently, the curves of the selected libraries should be lower than the one for the control library (Fig. 1b).

The insertion profile informed about the integration bias for the vector used, e.g., both transposons and retrovirus favor integration into transcription starting sites (TSS) in human and mouse genomes^9^,^29^,^30^,^31^,^32^ (Fig. 1c). If duplicated reads were not removed in the pre-processing steps, the preference for the TSS is masked due to PCR artifacts (see Supplementary Fig. S1); however, the effect of duplicates removal on final outcomes has not been investigated yet. We provide more details in the ‘Duplicates Removal’ section.

Finally, PCA plot helped to monitor the similarity between samples (Fig. 1d). Samples belonging to the same condition should be clustered together. Otherwise, potential confounding factors, like batch effects, must be investigated.

### Identification of blind spots

One of the main advantages of gene-trap screens, using haploid cells, is thought to be its ‘unbiased’ nature, as all vectors can be inserted into virtually all positions in the host genome. However, in previous studies, some integration preferences have been observed (‘hot spots’) for various transposons and retroviruses close to the TSS of expressed genes^29^,^30^,^31^,^32^. On the contrary, the ‘cold spots’ of these vectors in mammalian haploid cells have not been characterized. These genomic elements located in cold spot regions of the genome are excluded from the selection process, thus becoming the ‘blind spots’ of the screening experiment. Using both human and mouse datasets, we first identified the blind spots existing in both haploid genomes. Subsequently, we investigated potential contributing factors, and tested whether the elements could be recovered by increasing the sequencing depth or by introducing more replicates in our analysis. All following analyses were performed in the control libraries, as theoretically all insertions identified in the selected libraries are present in the control libraries.

**Figure 2:**
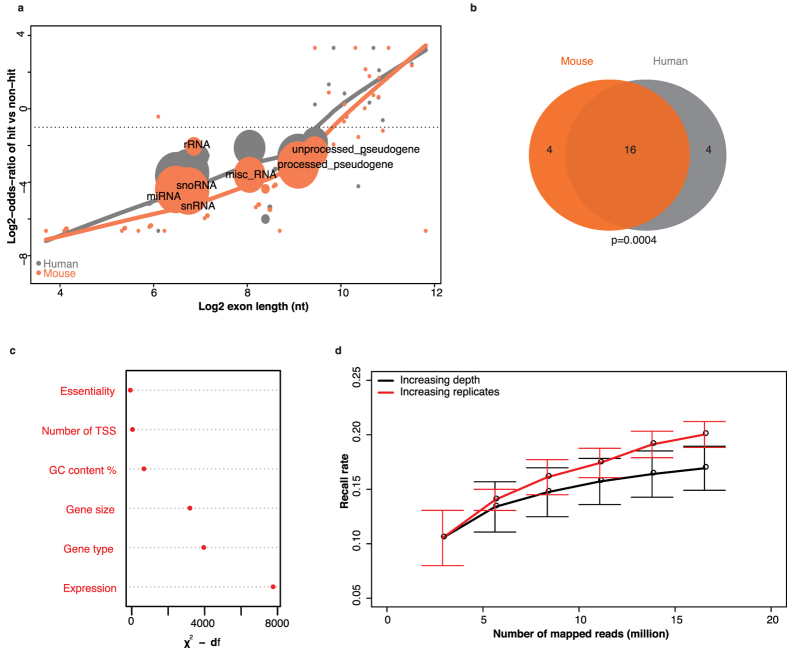
Blind spots of PhiT-seq. (**a**) Relation between gene size and enrichment of insertion in both human and mouse control libraries. Genes were first categorized into Ensembl gene types. For each gene type the log2-odds-ratio from Fisher’s exact test was plotted against its median gene size. In the test, a positive value means the gene type was enriched by genes getting inserted, and *vice versa*. Gene types conserved in human and mouse, as well as FDR < 0.05 and log2-odds-ratio < −1 (dashed line) in Fisher’s exact test, were plotted as dots, where the size was proportional to the extent of significance level (−log10-FDR). Other gene types were plotted as asterisks. The trend was fitted by LOcally WEighted Scatterplot Smoothing (LOWESS), in grey (human) and in orange (mouse) curves. (**b**) Gene types indicated as circles in (**a**) significantly overlapped in human and mouse (p: 0.0004 from Fisher’s exact test). (**c**) Factors were ranked according to the contribution to the insertion density (TPM) in the human dataset, where transcriptomic data are available. Essentiality, number of TSS (transcription starting site), GC content %, gene size, gene type and transcripts abundance (Expression, in TPM) were collected from published papers and databases, and a multiple regression model was constructed to assess the contribution (goodness of fit χ^**2**^ minus df) of each factor. df: degree of freedom (**d**) Saturation curves in the mouse dataset. For each control library, reads were included repeatedly, either from the same library (increasing sequencing depth) or another control library (increasing biological replicates). At each point, recall rate was calculated as the number of hit genes with given reads divided by the number of hit genes using all reads from all control libraries. Then, the median of the recall rate was calculated across all 6 replicates, together with median absolute deviation indicated as error bar.

A simple but direct explanation of blind spots is the smaller size of a genomic element, as shorter genes have fewer chances to get targeted. Instead of investigating individual genes, we performed the analysis in terms of gene types defined by Ensembl, aiming to provide a genomic view of the blind spots. For each gene type, the log2-odds-ratio of genes getting targeted versus not targeted was plotted against the gene size, as shown in Fig. 2a. Gene types with fewer insertions compared to random chance (FDR < 0.05 from Fisher’s exact test and log2-odds-ratio < −1) were highlighted. We observed several small genomic elements with few insertions, such as miRNA (microRNA), snRNA (small nuclear RNA), snoRNA (small nucleolar RNA) and rRNA (ribosomal RNA) (Fig. 2a). On the contrary, genes with larger size, such as protein coding genes and lincRNA (long intergenic non-coding RNAs) presented a high number of insertions (see Supplementary Table S1A,B). Meanwhile, two other types of genes (processed and unprocessed pseudogenes) represented another blind spots in PhiT-seq experiments (Fig. 2a). These genomic regions contained many repeated sequences and could be defined as low mappability regions. Indeed, reads with multiple-hits in the genome were discarded after alignment (see Materials and Methods). Interestingly, these patterns were partially conserved between human and mouse haploid cells (Fig. 2a). Among a total of 40 gene types common between human and mouse, 20 types were identified enriched by genes lacking insertions in each species, respectively, and 16 of them overlapped (Fig. 2b; see Supplementary Table S1C).

To further identify potential factors responsible for these blind spots, we established a regression model, including gene essentiality^4^, number of transcription starting sites (TSS), GC content (%), gene size, gene type and gene expression level for human HAP1 cells. This regression model was performed only on human data due to the availability of the gene expression level and essentiality information^4^ missing for mouse haploid cells. Results showed that gene size and type were important factors (Fig. 2c). As previously described^29^,^30^,^31^,^32^, we observed that the expression level contributed most to blind spots, after adjusting for all the other factors. This is probably linked to the chromatin state of unexpressed genes.

Subsequently, we assessed two approaches to circumvent these blind spots. The first approach was to increase sequencing depth, thus genes with rare insertions could have higher chance to be identified; the second was to introduce more biological replicates, accounting for the inherent stochasticity of vector insertion. As only the mouse dataset includes biological replicates (see Supplementary Table S2), the comparison was performed only for these PhiT-seq data^7^. By resampling the reads, we found that more replicates achieved higher coverage rates than more sequencing depths (Fig. 2d). These results were in line with what was observed in RNA-seq experiments^33^. However, the introduction of replicates also challenged the previous methods analyzing PhiT-seq data, namely Fisher’s exact and the binomial tests, due to the biological variance. Other methods accounting for this biological variance are discussed in the ‘Statistical analysis with biological replicates’ section.

### Recompiling annotation with transcriptomic data

Counting insertions for each gene is the basis for all downstream statistical analysis. Tools developed for RNA-seq, like HT-Seq^34^ and featureCounts^35^, count only reads in sense orientation of exons. They cannot be directly implemented in PhiT-seq, as an ‘effective’ insertion (EI) needs to be distinguished from its counterpart. An EI can be located in the promoter, splicing site, exon or sense orientation of an intron, as these insertions are assumed to have an influence on the transcription of a gene.

**Figure 3:**
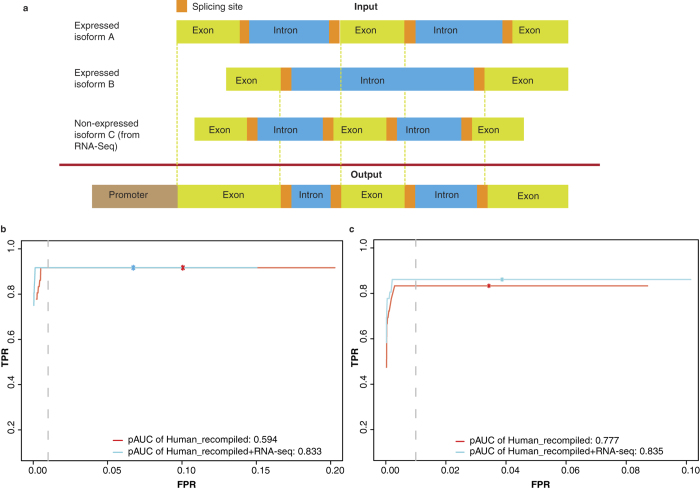
Gene annotation used in VISITs. (**a**) A conceptual gene model after integration with transcriptomic data. Assuming that this gene has three isoforms, two expressed, A and B, and one not expressed C. The recompiling process will combine the exons in A and B. Transcriptomic data, if available, can be integrated by removing non-expressed isoforms C. Promoter region could also be included. (**b**) Receiver Operating Characteristics (ROC) curves comparing different annotation methods in the human dataset, using Fisher’s exact test for the count enrichment. The red curve represents counting the insertions using the recompiled gene model, but without integration of RNA-seq data. The blue curve represents counting the insertions using the recompiled gene model, as well as RNA-seq data integrated, i.e., non-expressed isoforms removed, after quantification by Kallisto. Partial Area Under ROC Curve (pAUC) were calculated at FPR = 0.01. Asterisks were labeled at FDR = 0.01. (**c**) ROC curves comparing different gene models described in (**b**) in the human dataset, using the binomial test for the sense enrichment, with the same cutoff in (**b**).

Nowadays, due to existing time/tissue-specific splicing events in mammalian transcriptomes^36^, the definition of ‘exonic region’ remains elusive. To keep as much information as possible, we generated a conceptual gene model by combining all possible exons, as well as splicing sites and promoter regions (Fig. 3a). We also recompiled the gene model by integrating RNA-seq data (Fig. 3a). Original gene models (here from Gencode^37^) were recompiled by utilizing only expressed transcripts, plus canonical splicing sites (5′-end GU and 3′-end AG) and optionally, the promoter region. When RNA-seq data were not available, all annotated isoforms were used in order to keep all EIs. The advantage of using RNA-seq data is straightforward and can be justified by two arguments. In statistics, removing transcripts reduced the total number of tests, leading to an increase in the specificity of the analysis. Moreover, in biology, silent genes cannot play a role in the selection process, even though targeted by vectors. A similar strategy has been successfully implemented previously in RNA-seq data analysis, to improve the performance on the identification of differential splicing events^38^.

To illustrate the performance of the recompiled gene model, two tests were conducted in the human dataset (as no RNA-seq data are available for mouse haploid cells). In PhiT-Seq experiment, EIs should be enriched in relevant genes in the selected library compared to the control library. In this case, data are represented as count of EIs and therefore Fisher’s exact test can be used^6^,^39^. It is also assumed that the proportion of EIs in relevant genes should be increased after selection, as non-EIs cannot disrupt gene expression, thus cells carrying them get eliminated during the selection process. In this situation, data are represented as proportion of EIs (ratio between EI and total number of insertions for each gene) and the binomial test was used^4^. Both of these two tests were used in the original publications for the selection of candidate genes^10^,^40^. Importantly, these two tests can be only performed in single replicate and when biological replicates exist, other methods have to be used (see next section). Simply for convenience, we will refer to these two tests as ‘count enrichment test’ and ‘sense enrichment test’, regardless of the statistical methods used.

Thereafter, we evaluated the recompiled gene model in the human dataset for both count and sense enrichment test. Performance was measured by ROC (Receive Operating Characteristics) curves and rescaled pAUC (partial Area Under Curve) at false positive rate (FPR) = 0.01, using Fisher’s exact test for count enrichment and the binomial test for sense enrichment. Improved performances (pAUC: 0.833 vs 0.594 in count enrichment test and 0.835 vs 0.777 in sense enrichment test) were observed after integration with RNA-seq data (Fig. 3c,d). Finally, we confirmed that the improved pAUC did not happen by chance, using random filtering of the same number of non-expressed genes for 1,000 iterations (p < 0.001 for count enrichment test, and p = 0.024 for sense enrichment test, see Supplementary Fig. S2).

### Statistical analysis with biological replicates

When biological replicates exist, variance has to be taken into account in order for genes that are behaving consistently across replicates to rank higher than erratic genes^41^. For this study, we used the mouse dataset^7^, as it was the only accessible PhiT-seq dataset including biological replicates. For count data, the analysis was performed using the Wald test developed in DESeq2, where generalized linear model (GLM) is supported for complex experimental design^42^. For dispersion estimation, we evaluated several methods, including common and tagwise dispersion from edgeR^43^, Local-Fit methods from DESeq2^42^ and hierarchical Bayesian methods from DSS^44^. To exclude the potential influence of normalization methods, data were normalized to total count in all following comparison. Effects of normalization methods are evaluated in the ‘Normalization’ section. Performance was measured using ROC curves and a rescaled pAUC at FPR = 0.01.

**Figure 4:**
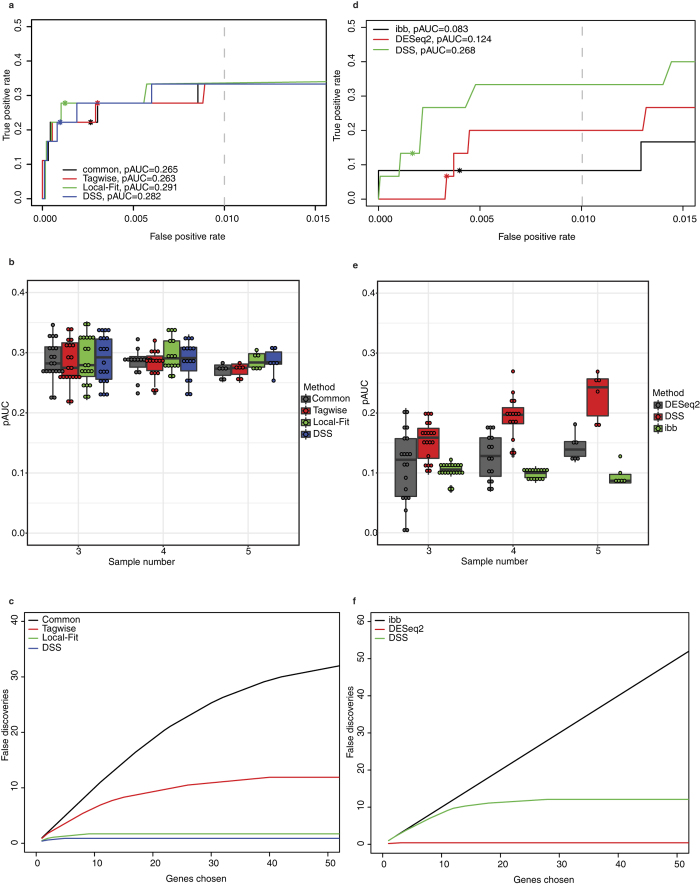
Comparison of different statistical methods to account for biological replicates in the mouse dataset. (**a**) Comparing different shrinkage methods in count enrichment tests using ROC curves for all samples. The curve was truncated at FPR = 0.01, where rescaled pAUC was calculated. Asterisk was labeled at FDR = 0.01. (**b**) Performance of different methods in count enrichment tests with different subset of samples (from 3–5) using pAUC. (**c**) False discovery curves within control samples for comparing different shrinkage methods in count enrichment test. Three samples from control libraries were labeled as ‘selected library’ and compared with the rest of the control libraries at FDR < 0.05. The curve showed the number of false discoveries after averaging all possible compositions. (**d**) Comparing different methods in sense enrichment test using ROC curves and pAUC, as (**a**). (**e**) Performance of different shrinkage methods in sense enrichment test with different subset of samples (from 3–6) using pAUC, as (**b**). (**f**) For sense enrichment test, false discovery curves were also compared using the same method, as (**c**).

As shown in Fig. 4a, all methods performed comparably, though Local-fit showed a slight improvement in pAUC. To further look into the performance on smaller sample size, another comparison was conducted using different number of replicates, ranging from 3 to 5 in each condition. Local-Fit and DSS showed consistently better performance in terms of pAUC when the sample size was 4 or 5, and comparable performance when the sample size was 3, compared to other methods (Fig. 4b). Subsequently, we tested the specificity of these methods by using only samples from the control libraries, reasoning that we should identify no genes by comparing samples within the same condition. Indeed, all genes with small FDR values were considered to be false discoveries. Local-Fit and DSS scored best compared to the other methods (Fig. 4c). Finally, we also compared the Wald test from DESeq2 with quasi-likelihood F-test from edgeR and Voom + Limma packages^45^,^46^ (see Supplementary Fig. S3). Generally, comparable performances were observed among these methods, with the Wald-test scoring highest in terms of pAUC.

For proportion data, similar comparisons were performed using DSS, DESeq2 and ibb^47^. As DESeq2 does not directly model beta-binomial data, it was implemented with an alternative method for testing an interaction term, i.e., whether EI depends on the cell type, by comparing the full model in equation (1) with the reduced model in equation (2):


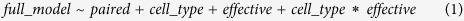



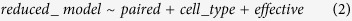


As shown in Fig. 4d, DSS showed superior performance compared to other methods in terms of ROC curves and pAUC. The superior performance was also consistent across different sample sizes (Fig. 4e). DESeq2 performed moderately in the comparisons, but it became highly conservative under a proper FDR cutoff (sensitivity < 0.1 at FDR = 0.01 in Fig. 4d). Moreover, in some comparisons with three replicates, its pAUC almost fell to zero (Fig. 4e, two dots around pAUC = 0). Although DSS demonstrated inferior false discovery control compared to DESeq2 (Fig. 4f), we still recommend this method for the sense enrichment test, as its sensitivity is higher than others. Based on these results, we used the Wald-test and Local-Fit for the count enrichment test and DSS for sense enrichment tests in the next analysis.

### Duplicates removal

Removing duplicates is a widely used practice to correct amplification bias when analyzing NGS data. However, in PhiT-seq data, there are no agreed strategies for removing duplicates. As shown in Supplementary Fig. S1, duplicates might arise from PCR artifacts, leading to unexpected noise. Therefore, in all analysis performed above, duplicates have been removed in the pre-processing step. However, duplicates could also derive from the expansion of clonal cells, which may carry a predominant insertion after selection. Here, we did not attempt to distinguish the origin of the duplicates, but instead tried to test the influence of different duplicates-handling strategies on the final outcome. We investigated the effect of different definition of duplicates on the statistical performance (starting at the same position in the same strand or with a difference up to 2 bp, as well as keeping all duplicates) in both control and selected libraries, across all scenarios, i.e., count/sense enrichment tests, and with/without replicates.

**Figure 5:**
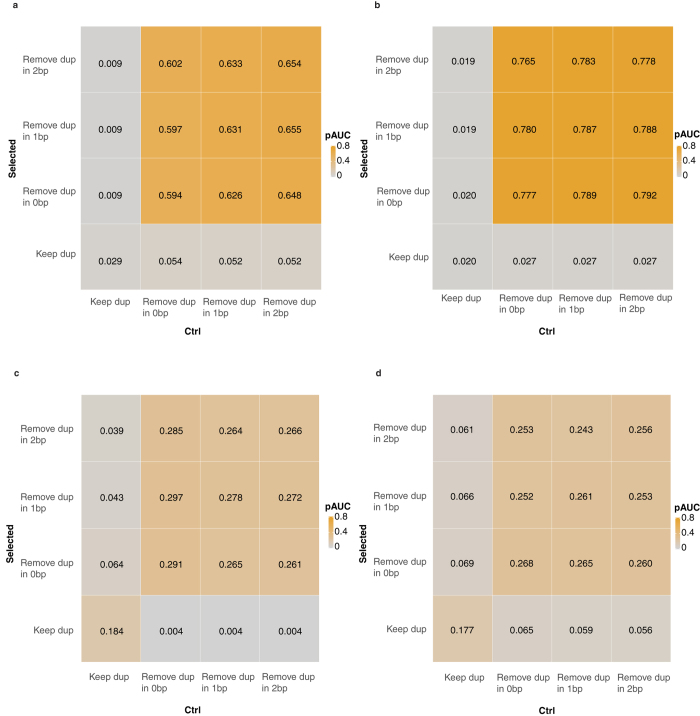
Comparison of the effects of duplicates removal on VISITs performance in human and mouse datasets. Rescaled pAUC were calculated at FPR = 0.01 with different definition of duplicates in control and selected libraries of the human dataset, using count (**a**) and sense enrichment (**b**) tests. Duplicates were defined as those have same insertion site (0 bp), within +/−1 bp, and +/−2 bp. Similar analysis was performed in the mouse dataset for count (**c**) and sense enrichments (**d**).

As shown in Fig. 5a–d, performance was greatly improved when duplicates were removed in all situations. More stringent definition (e.g., extending the definition of duplicates up to 1–2 bp) further improved the performance in most cases, though the effect was only minor. Moreover, we did not observe a consistent pattern in all situations, as most duplicates were located at the same site (see Supplementary Table S4), thus more stringent definition had fewer effects on the final outcome. Based on these results, we recommend the elimination of duplicates in both control and selected libraries at the pre-processing step.

### Normalization methods

Several normalization methods have been proposed for NGS data analysis to correct for potential bias, e.g., GC-content^48^ and compositional bias^49^; however, no consensus has been reached in the scientific community for PhiT-seq data. Consequently, we first evaluated whether the GC bias needed to be corrected and compared the performance of different normalization methods.

To investigate the GC bias, the number of insertions sites was plotted against the GC content of each gene (see Supplementary Figs S4–5). Intriguingly, we did not observe an apparent effect of GC bias on the readout. Moreover, the patterns were very similar between the controls and the selected libraries in human and mouse datasets. We concluded that PhiT-seq data are less vulnerable to GC bias, as duplicates are removed in the pre-processing step, thus PCR artifacts have less effects on counting insertion sites compared to counting total reads. Therefore, in all our analysis, we did not correct for the GC bias.

Afterwards, we compared four different methods, including total count, relative log expression (RLE)^42^, trimmed mean of M-values (TMM)^49^ and CisGenome^50^. Comparisons were performed in all scenarios using pAUC as performance index. Results showed that the RLE method outperformed all the other approaches in the situation of a single replicate and had comparable performance when multiple replicates were available (Table 1). Comparison of different sample sizes (from 3 to 5 replicates in the mouse dataset) (see Supplementary Fig. S6a,b) also showed comparable performance for all methods. RLE also represented the best false discovery control in both count and sense enrichment tests (see Supplementary Fig. S6c,d). Based on these results, RLE was used in the following sections and recommended in PhiT-Seq data analysis.

### Inclusion of promoter region

When recompiling the gene model, the promoter region can be included, considering that the insertion of a vector in this region can suppress or enhance gene transcription^9^,^51^. To assess the impact of promoter inclusion on the final outcome across all scenarios described above, we tested the inclusion of promoter region ranged from 0 to 5 kb upstream of the TSS (see Supplementary Fig. S7a,b). Results showed decreased pAUC curves in all tests. We assumed that the disruptive effect of insertions into upstream regions was not as influential as those in gene bodies, which most likely led to a truncated protein instead of a regulating effect. Therefore, including insertions in upstream regions will somehow compromise the results. However, the performance became stable after 3 kb, probably due to the few insertions found beyond this region (see Supplementary Fig. S1).

### Prioritization and verification

**Figure 6:**
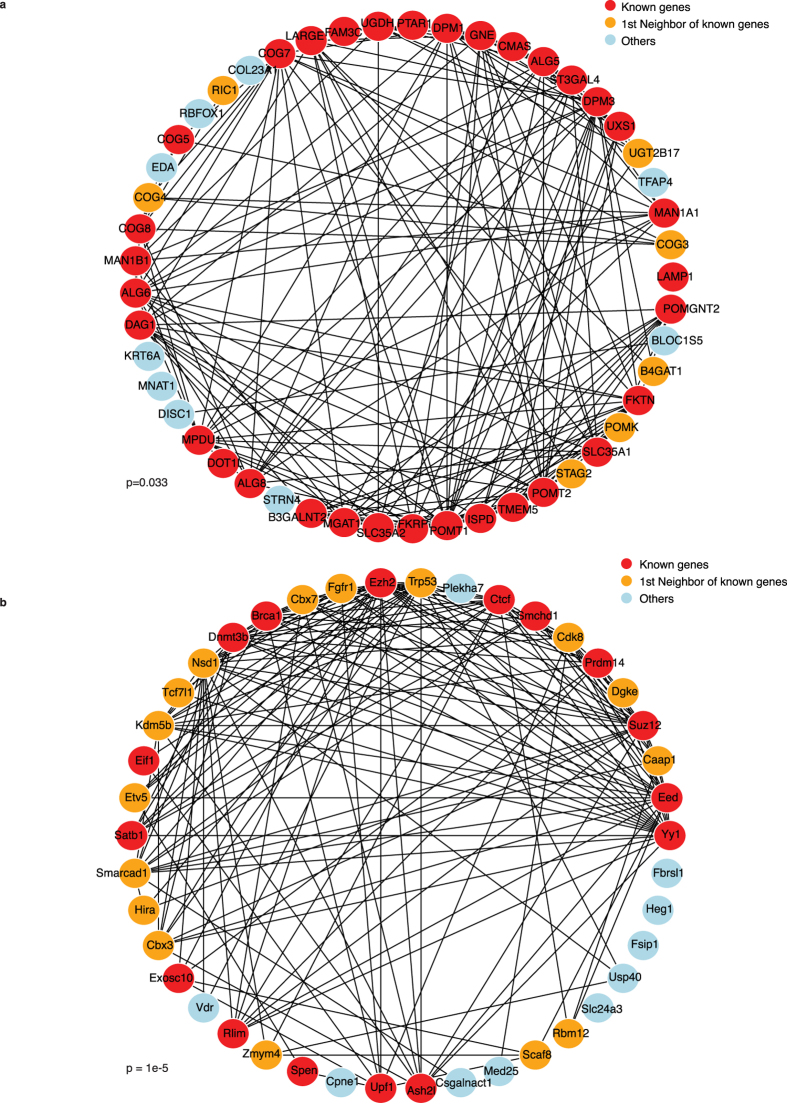
STRING network showing the interaction between known genes (red) and potential candidates. Potential candidates were defined as the top 30 candidates (excluding known genes) using combined FDR in the (**a**) human and (**b**) mouse datasets. 1^st^ order neighbors of known genes were shown in orange and high-order neighbors or isolated nodes were shown in blue. Empirical p-values were generated using a randomly permuted STRING network for 10,000 times, calculating the proportion of times where the summarized connectivity to the known genes from the novel candidates is larger than the observed one.

To rank the candidate list, we derived a combined score from both count and sense enrichment tests^52^. ROC curves and pAUC (see Supplementary Fig. S8a,b) showed enhanced performance on both datasets when using the combined score, compared to results using either the count or sense enrichment test alone. To illustrate the ability of the combined score to reveal novel candidates, we calculated the connectivity between known genes and novel candidates using the protein-protein interaction network defined in the STRING database^53^. In the human dataset, novel candidates revealed with the combined score were more connected to known targets compared to count or sense enrichment methods only (see Supplementary Fig. S8c). For the mouse dataset, better results were obtained by the count enrichment method and the combined score compared to the sense enrichment method only (see Supplementary Fig. S8d). The STRING networks incorporating known genes and 30 potentially novel candidates are presented in Fig. 6 for both datasets. The human study aimed to identify novel regulators of Lassa virus entry. *COG3* and *COG4* genes were identified as potential candidates (combined FDR: 2.54e-29 and 8.00e-29, respectively) and represented as direct neighbors to known genes in the network (Fig. 6a). Indeed, these two genes have been identified in another screening experiment, which aimed to detect genes involved in the infection of Rift Valley fever virus^19^. The mouse study attempted to reveal unknown regulators of the X chromosome inactivation process. The *Cbx7* gene was identified as a potential new candidate (combined FDR: 6.19e-7) and was also directly connected to known genes in the STRING network (Fig. 6b). *Cbx7*, a member of the polycomb complex 1, has been implicated in the XCI process previously^54^. Of note, *Cbx7* may not have been included using only sense enrichment test given its marginal FDR (0.057). Notably, these candidates were not identified in the original paper of these two datasets. The detailed candidate lists from our method and the original papers are provided in Supplementary Data 1–2 and compared in Supplementary Fig. S9.

### VISITs pipeline

**Figure 7:**
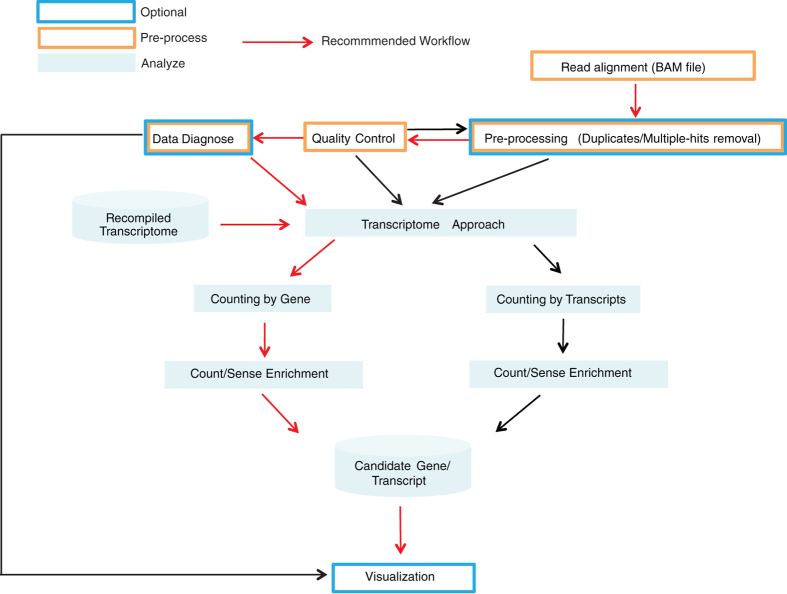
Workflow of VISITs. The input should be BAM files after alignment to the genome. After pre-processing and quality check, two different approaches could be used, 1) counting by gene: Insertions were counted in recompiled gene model; 2) counting by transcript: Insertion sites were counted in recompiled transcript model. RNA-seq data can be integrated if available in both approaches. An R markdown file was generated to visualize the results. The counting-by-gene approach is recommended.

The VISITs pipeline can be divided into several modules: pre-processing, QC, data diagnosis, statistical analysis and visualization, as shown in Fig. 7. BAM files from any aligner can be used as input files. VISITs will then perform pre-processing and QC in a first step. For data diagnosis, GC-content bias (see Supplementary Figs S4–5), intra-group variance (see Supplementary Fig. S10), and Minus-Average (M-A) plot (see Supplementary Fig. S11) will be generated. Results from QC and data diagnosis should be interrogated before performing statistical analysis (see Supplementary Table S5).

For statistical analysis, when RNA-seq data are available, it is recommended to use the transcriptomic approach, where non-expressed transcripts and genes are removed. Two methods of counting are available, i.e., counting insertion sites at the gene level or at the transcript level. Due to the lack of ground-truth evidence of interesting biological questions (here lassa virus entry and XCI) on the transcript level, results for these studies are presented at the gene level. In addition, transcriptome reconstruction remains challenging due to insufficient sequencing depth of RNA-seq data or due to the unavailability of data from mouse haploid cells. We also tested a sliding-window approach on these two datasets, but the performance obtained was very limited (data not shown), probably due to lower coverage of insertions (see Supplementary Table S3) and ignorance of the existing gene model.

Finally, VISITs produces an HTML report for results visualization with insertion tracks (see Supplementary Fig. S12) and a bubble plot summarizing top candidates (see Supplementary Fig. S13).

## Conclusions

Compared with other screening approaches, e.g., RNAi or CRISPR-Cas9 screens, PhiT-seq in haploid cells has two main advantages: no off-target effects have been reported and the coverage of genomic features is higher, including promoters and lncRNAs. In recent publications, comparable performances were also observed between these screening methods in identifying essential genes in yeast and human^4^,^5^.

One limitation of our study is that all EIs were treated equally in the counting process. However, an insertion located in a coding sequence (CDS) might be more influential than an insertion in the 5′/3′-untranslated (UTR) or enhancer. An ideal approach should consider the different effect of insertions present in the UTR and CDS. Nevertheless, in practice it remains very challenging to quantify such differences, due to the lower coverage in intergenic regions and a short list of verified candidates. Another limitation is how the quality measure and analyzing approaches proposed here could be generalized to other experiments. Indeed, available PhiT-seq data are still limited. With more PhiT-seq data becoming available, we expect to further refine the measures and approaches developed in VISITs.

VISITs is the first pipeline that is dedicated for pre-processing, QC, statistical analysis and results visualization of PhiT-seq data. In our study, we illustrated the usage of VISITs on two positive selection screens (identification of survival genes after selection). More importantly, although PhiT-seq experiments published before were mostly performed without replicates, we have noticed more published experiments with replicates in the recent years^4^,^7^. By using existing frameworks developed for other NGS data analysis, VISITs enables handling of biological variance as well as more complicated experiment design.

## Materials and Methods

### Human and mouse datasets

The human PhiT-seq dataset, used in our study, was retrieved from NCBI Sequence Read Archive under accession number SRP018361. In this dataset, SRR663777 was the control library and SRR656615 was the selected library^10^. The 36 genes used as ‘true-positive’ in our study were extracted from two publications^10^ (see Supplementary Table S4). The other two selected libraries undergoing DAG1 (SRR663778) and heparan sulfate depletion (SRR663779) were also used, but only in the quality control step. RNA-seq data from human HAP1 cells were retrieved from SRA under accession SRP044391. It includes two biological replicates sequenced by single-end protocol^20^.

The mouse PhiT-seq dataset, used in our study, was retrieved from NCBI Sequence Read Archive under accession number SRX1060416 (control) and SRX1060407 (selected). Six biological replicates (ELAM4/5/7/8/9/10) were retrieved from each accessions in a paired way^7^.

For detailed information about these two datasets, see Supplementary Table S2.

### Mapping

For the human PhiT-seq dataset^10^, reads were first trimmed to 36 nt, then mapped to hg38 using bowtie (v1.0.0)^24^, allowing no mismatches, as described in Jae *et al*.^10^. For the human RNA-seq dataset^20^, reads were mapped to hg38 using STAR (v2.4.2a)^55^, allowing up to 2 mismatches. For the mouse PhiT-seq dataset^7^, reads were first trimmed to remove adapters using trimmomatic (v0.32)^23^ and then mapped to mm10 using bowtie2 (v2.2.3)^56^, with default settings as described in Monfort *et al*.^7^.

### Pre-processing and quality control (QC)

Reads with multiple-hit positions or mapped on the chrY were removed. Reads with same starting genomic coordinate and orientation were collapsed to derive independent insertion sites. Both procedures were done using customized scripts, which are available in VISITs.

Bedtools (v2.22.0)^57^ were used to count the number of independent insertions in each gene, using Gencode^37^ human V23 and mouse M6 as gene models. For Manhattan plot, TPM (transcripts per million reads) were calculated for each gene, where the transcript length was replaced by gene size, as the insertions were counted in both intron and exon. For PCA plot, data were first transformed by function varianceStabilizingTransformation(), then visualized by plotPCA() in DESeq2 (v1.10.1)^42^. For saturation plot, function generateSubsampledMatrix() in subSeq (v1.0.1) was used to randomly sample the count matrix from 1% to 97% in a step of 3%. ggplot2^58^ was used to generate all the plots in QC module, except the coverage profile, which was generated using ngsplot^59^.

### Identification of blind spots

Genes were first divided into different types (protein coding genes, rRNAs, tRNAs, miRNAs, etc.) by Ensembl using biomaRt^60^. For each type, the number of genes without insertions (defined as no insertions in the control library) was compared with those happened by chance using Fisher’s exact test, generating log2-odds-ratio and p-values. All multiple testing corrections in our study were performed using Benjamini-Hochberg method^61^.

A linear model was established to estimate the contribution of following factors to the number of insertions (TPM) in each gene:

1. Gene Essentiality^4^;
2. Number of transcription starting sites (Number of TSS), generated from Gencode^37^;
3. GC content %, from biomaRt^60^;
4. Gene size, from Gencode^37^;
5. Gene type, from biomaRt^60^;
6. Expression Level from human HAP1 cells. Read counts for each gene were summarized using featureCounts (v1.4.5)^35^. Expression levels were defined as the average TPM of the two replicates.

Number of independent insertions in each gene was first log2-transformed, and fit by function ols in rms^62^, using restricted splines to account for non-linear effects and Akaike information criterion to decide the usage of degree of freedom.

### Recompiling gene models

The number of independent insertion for each gene was counted and compared using two different approaches:

1. Recompiled Model: All potential exons together with canonical splicing sites (i.e., 2 nt at both ends of intron) were concatenated, forming a conceptual transcript. The remaining part of the gene was considered as intronic region. Bedtools^57^ (v2.22.0) was used to count the insertions for exon and intron in a strand specific way.
2. Recompiled Model with RNA-seq data: Kallisto (v0.42.1)^63^ was used to quantify the transcripts. The transcripts with positive TPM (Transcripts Per Million reads) were kept, and concatenated to form a conceptual transcript.

In both approaches R package genomeIntervals (v1.26)^64^ was used.

### Handling biological replicates

For count enrichment, Voom^45^, DESeq2^42^, edgeR^43^ and DSS^65^ were compared. In edgeR, function estimateDisp() was used; in DESeq2, function estimateDispersions() with Local-Fit was used. When implementing DSS, function estDispersion() was used to replace the previous two functions in edgeR and DESeq2. For sense enrichment, DESeq2^42^ was also used with default dispersion estimation methods.

For all comparisons, data were normalized to total count first. pAUC (partial Area Under ROC Curve) was calculated at false positive rate 0.01, using R package ROCR^66^. Independent filtering and outlier detections were disabled in edgeR and DESeq2, as we focused on comparing the approaches on dispersion estimation. All tests were performed as one-sided.

### Normalization

Four normalization methods were compared: total count, RLE (used in DESeq2^42^), TMM (used in edgeR^43^) and adapted CisGenome (used in ChIP-Seq^50^). For CisGenome, the basic unit of counting windows was replaced by gene. For RLE and TMM, function calcNormFactors() in edgeR^43^ and estimateSizeFactors() in DESeq2^42^ were used with default setting, respectively.

### Parameter choice

Duplicates were removed using a customized script (pre-processing.sh) provided in VISITs. Insertions located in both sense/antisense strands of promoter regions were counted as EIs.

### Prioritization and verification

P-values from count and sense enrichment tests were combined using a weighed Z-test^52^, where the weights were the mean cpm (count per-million reads) across all libraries for count and enrichment tests. For genes without p-value from sense enrichment (e.g., the sense enrichment test cannot be performed on genes with only one exon), the combined p-value was the p-value from count enrichment.

STRING network^67^ was used to verify the new candidates. The networks were visualized using igraph (v1.0.1)^68^.

### Availability

VISITs is available at https://sourceforge.net/projects/visits/under GPL license.

## Additional Information

**How to cite this article**: Yu, J. and Ciaudo, C. Vector Integration Sites Identification for Gene-Trap Screening in Mammalian Haploid Cells. *Sci. Rep.* **7**, 44736; doi: 10.1038/srep44736 (2017).

**Publisher's note:** Springer Nature remains neutral with regard to jurisdictional claims in published maps and institutional affiliations.

## Supplementary Material

Supplemental Figures

Supplemental Tables

Supplemental Datset 1

Supplemental Datset 2

## Figures and Tables

**Table 1:** Performance of four normalization methods in different scenarios.

pAUC	count.Human	sense.Human	count.Mouse	sense.Mouse
Total Count	0.594	0.777	0.291	0.268
RLE (DESeq2)	0.790	0.806	0.286	0.281
TMM (edgeR)	0.580	0.780	0.268	0.277
CisGenome	0.592	0.776	0.283	0.278

Performance was measured using rescaled partial area under curve (pAUC) at false positive rate 0.01. Higher pAUC indicates better performance. count.Human: count enrichment test without replicates using fisher-exact test in the human dataset; sense.Human: sense enrichment test without replicates using the binomial test in the human dataset; count.Mouse: count enrichment test with biological replicates using DESeq2 in the mouse dataset; sense.Mouse: sense enrichment test with replicates using DSS in the mouse dataset. RLE: Relative Log Expression; TMM: Trimmed mean of M-values.
