# Supplementary material for: Vector Integration Sites Identification for Gene-Trap Screening in Mammalian Haploid Cells
Source: Sci Rep. 2017 Mar 17;7:44736. doi: 10.1038/srep44736 (PMC5356192; doi:10.1038/srep44736)
Supplement: Supplemental Figures [file srep44736-s1.doc]

Supplementary Figures for

Vector Integration Sites Identification for Gene-Trap Screening in Mammalian Haploid Cells

Jian Yu^1,2^ and Constance Ciaudo^1,*^

^1^Swiss Federal Institute of Technology Zurich, Department of Biology, Institute of Molecular Health Sciences, Chair of RNAi and Genome Integrity, Zurich, Switzerland.

^2^Life Science Zurich Graduate School, Molecular and Translational Biomedicine program, University of Zurich, Zurich, Switzerland.

^*^ To whom correspondence may be addressed: Prof. Constance Ciaudo.

E-mail: [cciaudo@ethz.ch](mailto:cciaudo@ethz.ch)

**Figure S1, Insertion profile near transcription starting sites (TSS) with or without removing duplicates,** **related to figure 1c.**

**(a)** For the mouse dataset^1^, six independent biological replicates were merged and insertion profiles were generated before and after removing duplicates. **(b)** For the human dataset^2^, insertion profiles were generated before and after removing duplicates for each sample. All insertion profiles were generated using ngs.plot^3^.

**Figure S2, Distribution of change of pAUC (indicated as ΔpAUC) after randomly removing 5,154 genes from the annotation file in the human dataset**^2^**.**

The black curve represents the count enrichment and the red curve the sense enrichment. ΔpAUC, generated by removing real non-expressed genes, were labeled in the horizontal axis. Permuted-P was calculated as the number of times that permuted ΔpAUC bigger than real ΔpAUC, divided by 1,000.

**Figure S3, pAUC for count enrichment test in the mouse dataset using different statistical tests and shrinkage methods.**

Rescaled pAUC were calculated at FPR=0.01 to compare different shrinkage methods for count enrichment test in mouse dataset^1^. Methods include common dispersion, local fitting, tagwise dispersion and DSS, using DESeq2 (wald test)^4^ and edgeR (quasi-likehood F-test)^5,6^. Voom+Limma^7,8^ packages were also included. Comparisons were performed when sample size increases from 3 to 5.

**Figure S4, GC-content bias in human dataset.**

Log2-transformed number of EIs (normalized against total count) against GC-content (%) for **(a)** the control library and **(b)** the selected library, using R package cqn^9^.

**Figure S5, GC-content bias in mouse dataset.**

Log2-transformed number of EIs (normalized against total count) against GC-content (%) for **(a, b, c, d, e, f)** control libraries and **(g, h, i, j, k, l)** selected libraries, using R package cqn^9^.

**Figure S6, Comparing different normalization methods in mouse dataset.**

Rescaled pAUC was calculated at FPR=0.01 for comparing different normalization methods for count enrichment test **(a)** and sense enrichment test **(b)** in mouse dataset^1^. Normalization methods include total count (TC), RLE (from DESeq2)^4^, TMM (from edgeR)^10^ and adapted CisGenome^11^. Comparisons for pAUC were performed when sample size increases from 3 to 6. The values of pAUC for all 6 samples correspond to those in Table 1. False discovery curves were generated for count **(c)** and sense enrichment tests **(d)**, respectively. Three samples from control libraries were labeled as ‘selected library’ and compared with the rest of the control libraries at FDR < 0.05. The curve showed the number of false discoveries after averaging all possible compositions of the 3-versus-3 comparisons.

**Figure S7, Comparison of the effect of upstream inclusion on VISITs performance in human and mouse datasets.**

Rescaled pAUC at FPR=0.01 was calculated with different size of upstream region included in the human^2^ (**a**) and mouse^1^ (**b**) datasets.

**Figure S8, Combined FDR achieved comparable performance for known genes, and generated more potential candidates, compared to FDR derived from count or sense enrichments, individually.**

**(a-b)** Performance of combined FDR (green curve) versus FDR derived using count (black curve) or sense enrichment (red curve) in human **(a)** and mouse datasets **(b)**. In both datasets, ROC curve and rescaled pAUC (at FPR=0.01) were generated. Asterisk was labeled at FDR=0.01. **(c-d)** Ability of combined FDR (green curve) to reveal new candidates, compared with FDR derived from count (black curve) or sense enrichment (red curve), individually, in human **(c)** and mouse datasets **(d)**. In both datasets, empirical p-values were generated from 10 to 50 potential candidates, using a randomly permuted STRING^12^ network for 10,000 times, calculating the proportion of times where the summarized connectivity between the known genes and novel candidates is larger than the observed one. If the empirical p-value is 0, it is set to 1e-5. Potential candidates were defined as the top 10 to 50 candidates ranked by FDR (excluding known genes). The gray line indicates an empirical p-value at 0.05.

**Figure S9, Comparison of results generated by VISITs and those in the two original papers.**

To investigate the difference our results with those already published, the human dataset^2^ was compared using the true-positive genes, as shown below. Improved power by our approaches can be seen in both count and sense enrichment methods. The same comparison cannot be performed for the mouse dataset^1^, as the author did not provide a full gene-list. However, we noticed in the Table S3^1^, where 25 significant candidates were listed. However, in this table, *Tsix*^13^, a well-known antisense lncRNA involved in X chromosome inactivation (XCI) was missing. Other possible missing XCI factors include *Suz12*, a subunit of polycomb complex^14^ and *Rlim*, a ubiquitin ligase^15^. These missing genes may indicate inadequate power of the original methods used in the mouse dataset^1^.

Boxplot of confidence level (indicated as log-transformed FDR) for 36 true-positive genes, using original gene-list generated in the human dataset^2^ (red boxes) and re-analyzed by our methods (blue boxes), for count and sense enrichments, respectively.

**Figure S10, Boxplot of intra-group variance of mouse dataset in selected and control libraries.**

For count enrichment, numbers of EIs were first normalized to total count. Subsequently, biological coefficient variances (BCV) were calculated using R function estimateTagwiseDisp in edgeR^5^, for control and selected libraries, separately. For sense enrichment, a standard deviation of proportion of EIs in each gene was calculated for control and selected libraries, separately. In both case, the intra-group variance should be smaller in control compared to selected libraries, due to random selection.

**Figure S11, Minus-Average plot for the mouse dataset**^1^**.**


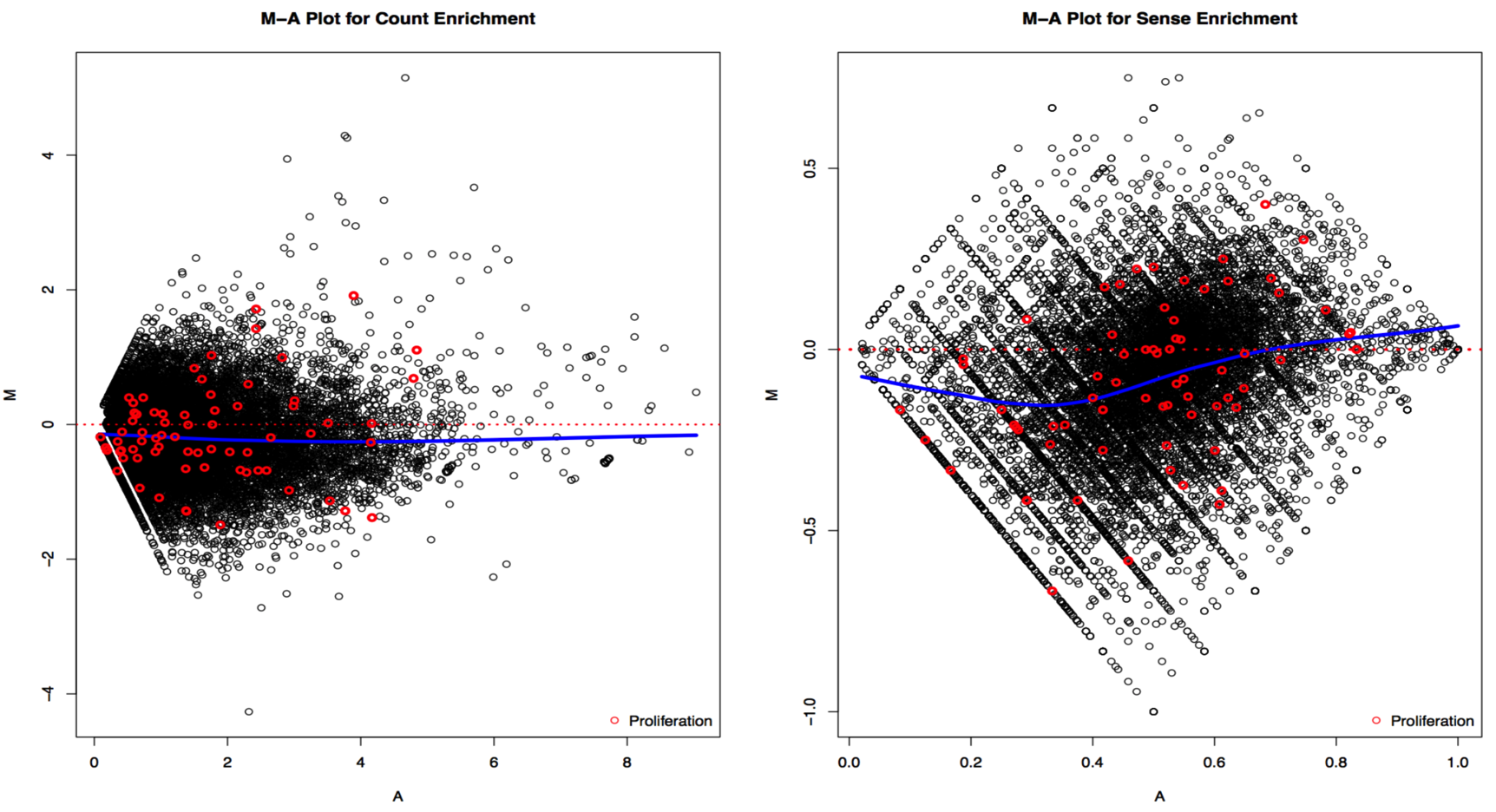


**(a)** For the count enrichment in the mouse dataset, independent insertions of each gene were first normalized to total count and then M (minus of selected vs Ctrl libraries) was plot against A (average of selected and Ctrl libraries). **(b)** For the sense enrichment in the mouse dataset, M of proportion of EIs was plot against A. For both figures, genes involved in regulation of proliferation in stem cells (GO: 200648) are highlighted in red.

**Figure S12, Coverage tracks of a known gene in the human dataset**^2^**.**

Coverage tracks of human gene *ST3GAL4* were generated using R package GenomeGraphs^16^ for control library (red) and selected library (black) in a strand-specific way. This gene was selected as an example for visualization as it has higher coverage and has been reported to be involved in Lassa virus infection in a second paper from the same group^17^. Insertions were observed enriched in exonic and sense strands. Gene model and chromosome coordinates were shown in bottom.

**Figure S13, Bubble plot in the mouse dataset**^1^**.**

Only the first 1000 genes ranking by combined FDR were shown in the plot, and the top 20 genes were highlighted. The y-axis indicates the significance level (-log10-transformed FDR); the a-axis indicates the chromosome and the size of the gene is proportional to the number of insertions.

**References**

1. Monfort, A., et al., *Identification of Spen as a Crucial Factor for Xist Function through Forward Genetic Screening in Haploid Embryonic Stem Cells.* Cell reports, 2015.

2. Jae, L.T., et al., *Deciphering the glycosylome of dystroglycanopathies using haploid screens for lassa virus entry.* Science, 2013. **340**(6131): p. 479-483.

3. Li, S., et al., *ngs.plot: Quick mining and visualization of next-generation sequencing data by integrating genomic databases.* BMC Genomics, 2014. **15**(1): p. 284.

4. Love, M.I., W. Huber, and S. Anders, *Moderated estimation of fold change and dispersion for RNA-seq data with DESeq2.* Genome Biol, 2014. **15**(12): p. 550.

5. Robinson, M.D., D.J. McCarthy, and G.K. Smyth, *edgeR: a Bioconductor package for differential expression analysis of digital gene expression data.* Bioinformatics, 2010. **26**(1): p. 139-140.

6. Lund, S.P., et al., *Detecting Differential Expression in RNA-sequence Data Using Quasi-likelihood with Shrunken Dispersion Estimates.* Statistical Applications in Genetics and Molecular Biology, 2012. **11**(5).

7. Ritchie, M.E., et al., *limma powers differential expression analyses for RNA-sequencing and microarray studies.* Nucleic Acids Research, 2015. **43**(7).

8. Law, C.W., et al., *voom: Precision weights unlock linear model analysis tools for RNA-seq read counts.* Genome Biol, 2014. **15**(2): p. R29.

9. Hansen, K.D., R.A. Irizarry, and Z.J. Wu, *Removing technical variability in RNA-seq data using conditional quantile normalization.* Biostatistics, 2012. **13**(2): p. 204-216.

10. Robinson, M.D. and A. Oshlack, *A scaling normalization method for differential expression analysis of RNA-seq data.* Genome Biol, 2010. **11**(3): p. R25.

11. Ji, H., et al., *An integrated software system for analyzing ChIP-chip and ChIP-seq data.* Nat Biotechnol, 2008. **26**(11): p. 1293-300.

12. Szklarczyk, D., et al., *STRING v10: protein-protein interaction networks, integrated over the tree of life.* NAR, 2015. **43** (Database issue): p. D447-52.

13. Lee, J.T., L.S. Davidow, and D. Warshawsky, *Tsix, a gene antisense to Xist at the X-inactivation centre.* Nature Genetics, 1999. **21**(4): p. 400-404.

14. Schoeftner, S., et al., *Recruitment of PRC1 function at the initiation of X inactivation independent of PRC2 and silencing.* Embo Journal, 2006. **25**(13): p. 3110-3122.

15. Shin, J., et al., *RLIM is dispensable for X-chromosome inactivation in the mouse embryonic epiblast.* Nature, 2014. **511**(7507): p. 86-U443.

16. Bullard, S.D.a.J., *GenomeGraphs: Plotting genomic information from Ensembl. R package version 1.32.0.* Bioconductor, 2016.

17. Jae, L.T., et al., *Lassa virus entry requires a trigger-induced receptor switch.* Science, 2014. **344**(6191): p. 1506-1510.
